# A population-based study on safety and health of drinking *Litsea coreana* tea (hawk tea)

**DOI:** 10.3389/fpubh.2025.1588368

**Published:** 2025-06-16

**Authors:** Han Zhang, Xiyu Pu, Haijun Huang, Qin Huang, Bao Yu, Weiguo Cao, Yong Zhang

**Affiliations:** ^1^College of Public Health, Chongqing Medical University, Chongqing, China; ^2^College of Traditional Chinese Medicine, Chongqing Medical University, Chongqing, China; ^3^College of Chinese Materia Medica, Chongqing University of Chinese Medicine, Chongqing, China; ^4^The College of Traditional Chinese Medicine, Chongqing Medical University, Chongqing, China

**Keywords:** hawk tea, food safety, population survey, lipid metabolism, cross-sectional study

## Abstract

**Background:**

A herbal tea of leaves and stems of *Litsea coreana*, named Hawk tea locally, has been traditionally consumed in some places in southwestern China. However, there is no human based evidence about the safety and health effects of drinking hawk tea. This study aims to illustrate possible healthy effects of consuming hawk tea in a population with a habit of drinking hawk tea in Chongqing city.

**Methods:**

A hawk tea producing area located in Chongqing city was selected as the study setting, and residents were randomly designated. Self-designed questionnaires were administered to collect data, including basic information, the behaviors of consumption of hawk tea, and various health information.

**Results:**

Among the 4,363 respondents, 2,278 (52.2%) drank tea more 1 time per month and 957 (42.0%) were heavy drinkers. Among 2,278 Hawk tea drinkers, 62 reported various symptoms after drinking, and logistic regression showed no relationship between the symptoms reported and the characteristics of consumption (duration, frequency, and volume) (*p* > 0.05). In addition, heavy hawk tea drinkers had a lower risk of hyperlipidaemia than light hawk tea drinkers (*p* < 0.001, 95% odds ratio = 0.26).

**Conclusion:**

With the long history of consumption in the population and the effects on health as were shown in this study, hawk tea poses no detectable health risks to consumers and shows some benefit to metabolism of lipids. It can be recognized and recommended as a safe and healthy herbal beverage for people to consume in daily life. Further interventional studies on its safety and health benefits are warranted.

## 1 Introduction

Hawk tea is made from *Litsea coreana* Levl. var. *lanuginosa*, a perennial, indeciduous, broad-leaved tree which is different from *Camellia sinensis* tree (green tea) (1–3). This wild plant is mainly distributed in mountainous areas of southwest China, such as Chongqing, Sichuan, and Hubei (4, 5). Drinking hawk tea has a long history, and the earliest records of hawk tea consumption can be traced back to the Ming Dynasty. It is believed that hawk tea has the advantageous of “quench thirst, brighten-eyes, strengthen the stomach, detoxification, and storage stable” (6, 7). The buds or leaves of *L. coreana* Levl. var. *lanuginosa* are collected and processed in a way like green tea, with the steps of fixing, rolling, and frying. The soup of hawk tea is amber in color, with a mellow and refreshing taste, sweet aftertaste, and the unique aroma of camphor plant. Hawk tea was popular in rural area of southwest China in past and now is extending to cites gradually. It was reported that current drinkers of hawk tea were more than 30 million in China (8).

Modern pharmacological studies have shown that flavonoids, polysaccharides, polyphenol, saponins, and volatile oils are the major components of hawk tea. Among which, flavonoids are the most abundant active ingredients in *L. coreana* leaves. In a review of Xuejing Jia and Ming Yuan, at least 29 monomeric compounds and corresponding structures were identified (8), including Hypericin, Isoquercitrin, Kaempferol-3-*O*-β-d-galactoside, Quercetin, Echinacoside, Quercetin, Kaempferol. The total flavonoids in dried hawk tea leaves range around 10.48–22.30 mg/g (9). Flavonoids have various health benefits. Xiao et al. (10) found that *L. coreana* leaves have a strong antioxidant capacity and can effectively scavenge DPPH (2,2-Diphenyl-1-picrylhydrazyl) free radicals. In addition, the flavonoid components in *L. coreana* leaves had significant protective effects on the liver injury from chemicals by Hu et al. (11) and Xu et al. (12). In addition, *L. coreana* leaves has been found to have hypoglycaemic, hypolipidemic, and hypocholesterolaemia effects in animals. For example, Chen et al. (13) showed that *L. coreana* leaf extracts can reduce the activity of *α*-glucosidase and lipase at certain concentrations to achieve hypoglycaemic and hypolipidemic effects (14–16). *Litsea coreana* leaves contain minerals, amino acids, vitamins, alkaloids, and other nutrients (17–19). These studies implies that drinking hawk tea may have various health benefits, which makes hawk tea a promising health beverage against those widely spreading chronic diseases.

Although there is a long history of hawk tea drinking in some areas of southwest China and sparse animal studies had been conducted to elaborate the possible health effects and related mechanisms, the direct evidence for human populations with drinking habit is absent when considering to promote hawk tea consumption as a function beverage in a wider population. Therefore, we investigated a population with drinking habit of hawk tea to further assess its safety and health effects.

## 2 Materials and methods

### 2.1 Study site

Wuxi County, located in western China, as a major producer of hawk tea has a tradition of consuming hawk tea. The population in Wuxi County is approximately 380 thousand population, and 5 out of 19 towns were randomly selected as investigation sites.

### 2.2 Study participants

Residents aged >18 years were eligible as participants, except for those who suffered from major mental diseases or intellectual disabilities that prevented normal communication. All the respondents provided informed consent to participate. This study was approved by the Ethics Committee of Chongqing University of Chinese Medicine (2023005).

### 2.3 Contents of the questionnaire

The questionnaire included basic information about the respondents (sex, age, height, weight, duration of residence, occupation, education level, smoking consumption, and alcohol consumption), current habits of hawk tea intake (duration, frequency, and amount of consumption), various adverse reactions after drinking hawk tea, and current health conditions.

### 2.4 The conducting of survey

**Figure 1:**
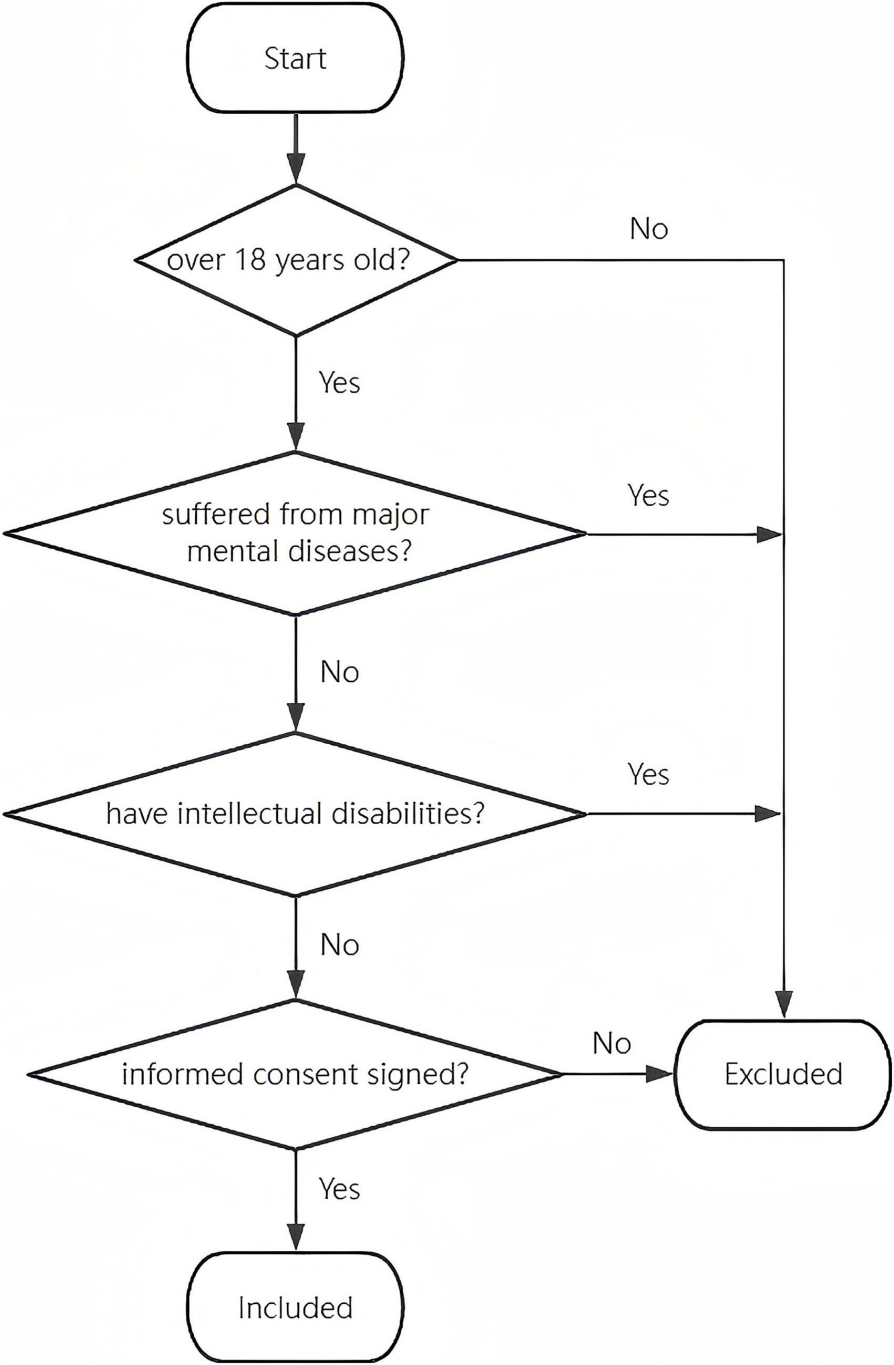
Participant selection process.

The survey was conducted in the summer of 2023, and the enumerators were trained to standardize its implementation. Subsequently, enumerators visited residents’ houses individually along the streets of the selected towns in one direction without returning, and eligible members of the household were invited to participate in the survey. Questionnaires were administered via face-to-face interviews to individuals who accepted the invitation. Figure 1 showed the procession of selecting the participants.

### 2.5 Data analysis

Questionnaire data were entered into Epidata 3.1 with a double entry. SPSS27.0 was used for statistical analysis. Descriptive analyses were performed using means and standard deviations for quantitative data and inter-group comparisons were performed using *t*-tests. The rates and constitutive ratios for categorical data and comparisons were calculated using the chi-squared test. The OR (odds ration) and its 95% CI (confidence intervals) between hawk tea consumption expressed as category variable and health outcome such as adverse reaction and chronic disease expressed as binary variable were estimated by using logistic regression. A *p*-value <0.05 was considered to be statistically significant.

## 3 Results

### 3.1 Characteristics of participants

In total, 4,663 persons accepted our invitation, 4,363 (93.6%) provided sufficient information for the analysis. Among them, 2,147 (49.2%) were male and 2,216 (50.8%) were female, and the mean age was 48.9 ± 16.1 years. The largest proportion of age was 18–45 years old (41.6%). The average BMI was 23.1 ± 3.9 kg/m^2^, with more than half (2,439, 55.9%) of the population having a BMI in the normal range. Farmer was the largest group of the survey respondents (42.1%), and their education level was predominantly primary school and below (40.6%). The prevalence of current smoking was 25.1% and current alcohol consumption was 12.9%. Of the nine common chronic diseases, hypertension had the highest prevalence of 12.8% in the total population, and cancer had the lowest prevalence of 0.9% in the total population.

Among the included respondents, 2,278 (52.2%) reported having a habit of drinking hawk tea (at least once per month). People with or without a habit of hawk tea drinking differ according to sex, height, weight, body mass index (BMI), age, duration of residence, education level, occupation, smoking habits, alcohol drinking, and hypertension. The characteristics of the respondents are presented in Table 1.

**Table 1:** Basic characterization of respondents.

Characteristics	Habit of hawk tea drinking^a^		Total	*F*/*χ*^2b^	*p*-value
	Yes (*N* = 2,278)	No (*N* = 2085)			
Sex				27.191	<0.001
Male	1,207 (53.0)	940 (45.1)	2,147 (49.2)		
Female	1,071 (47.0)	1,145 (54.9)	2,216 (50.8)		
Height (cm)	162.0 ± 7.4	161.4 ± 7.3	161.7 ± 7.3	0.251	0.005
Weight (Kg)	60.9 ± 11.2	59.6 ± 11.1	60.3 ± 11.1	0.983	<0.001
BMI	23.2 ± 3.8	22.9 ± 4.0	23.1 ± 3.9	0.984	0.009
BMI subgroup				13.161	0.004
Slimmer	120 (5.3)	156 (7.5)	276 (6.3)		
Normal	1,256 (55.1)	1,183 (56.7)	2,439 (55.9)		
Overweight	691 (30.3)	574 (27.5)	1,265 (29.0)		
Obesity	211 (9.3)	172 (8.2)	383 (8.8)		
Age	50.4 ± 15.8	47.2 ± 16.3	48.9 ± 16.1	7.443	<0.001
Age subgroup				45.367	<0.001
18–45	840 (36.9)	977 (46.9)	1817 (41.6)		
45–59	779 (34.2)	619 (29.7)	1,398 (32.1)		
>59	659 (28.9)	489 (23.5)	1,148 (26.3)		
Duration of residence in Wuxi County (year)				66.728	<0.001
<5	55 (2.4)	129 (6.2)	184 (4.2)		
5–10	68 (3.0)	99 (4.7)	167 (3.8)		
10–20	217 (9.5)	246 (11.8)	463 (10.6)		
20–29	351 (15.4)	352 (16.9)	703 (16.1)		
>29	1,587 (69.7)	1,259 (60.4)	2,846 (65.3)		
Educational level				9.609	0.008
Primary and below	967 (42.4)	803 (38.5)	1770 (40.6)		
Senior high school	425 (18.7)	378 (18.1)	1,272 (29.1)		
Above high school	886 (38.9)	904 (43.4)	1,321 (30.3)		
Occupation				22.325	<0.001
Farmer	1,034 (45.4)	805 (38.6)	1834 (42.1)		
Worker	193 (8.5)	181 (8.7)	374 (8.6)		
Civil servant	481 (21.1)	494 (23.7)	975 (22.3)		
Self-employed	129 (5.7)	130 (6.2)	259 (6.0)		
Service Industry	144 (6.3)	145 (7.0)	289 (6.6)		
Other occupation	297 (13.0)	330 (15.8)	627 (14.4)		
Smoking^c^				20.439	<0.001
No smoking	1,480 (65.0)	1,484 (71.2)	2,964 (68.0)		
Previous smoking	144 (6.3)	87 (4.2)	231 (5.3)		
Always smoking.	607 (26.6)	486 (23.3)	1,093 (25.1)		
Alcohol consumption^d^				23.242	<0.001
No drinking	1,302 (57.2)	1,304 (62.5)	2,606 (59.7)		
Sometimes	487 (21.4)	422 (20.2)	909 (20.8)		
Previous drinking	114 (5.0)	72 (3.5)	186 (4.3)		
Always drinking	309 (13.6)	256 (12.3)	565 (12.9)		
Chronic disease					
Hypertension	319 (14.8)	243 (11.7)	562 (12.9)	5.352	0.021
Diabetes mellitus	64 (2.0)	46 (2.2)	110 (2.5)	1.612	0.204
Hyperlipidaemia	68 (3.0)	52 (2.5)	120 (2.8)	0.981	0.322
Lung disease	45 (2.0)	29 (1.4)	74 (1.7)	0.073	0.788
Stroke	23 (1.0)	18 (0.9)	41 (0.9)	0.250	0.617
Heart disease	23 (1.0)	31 (1.5)	54 (1.2)	2.028	0.154
Liver disease	18 (0.8)	15 (0.7)	33 (0.8)	2.231	0.135
Cancer	8 (0.4)	6 (0.3)	14 (0.3)	0.137	0.711
Kidney disease	8 (0.4)	10 (0.5)	18 (0.4)	0.437	0.509

^a^Continuous variables were expressed using mean ± standard deviation and categorical variables were expressed as number of cases (percentage).

^b^Differences between continuous variables are tested by the ANOVA test and differences between categorical variables are tested by the chi-square test.

^c^Seventy-five people did not provide smoking information.

^d^Ninety-seven people did not provide alcohol consumption information.

### 3.2 Drinking behaviors of hawk tea drinkers

Among the 2,278 participants with the habit of drinking hawk tea (at least once per month), Heavy drinkers (those who had consumed hawk tea for >10 years, with a frequency of >3 times per week and >2 cups per serving) accounted for 42% of all drinkers. Heavy drinkers were more likely to be male, older, with longer duration residence in local, lower education, farmer, smoker, alcohol drinker, and lower proportion of hypertension and diabetes (all *p* < 0.05) (Table 2).

**Table 2:** Consumption characteristics of Hawk tea drinking.

Characteristics	Light drinkers (*N* = 1,321)	Heavy drinkers^a^ (*N* = 957)	*χ* ^2^	*P*
Gender			18.035	<0.001
Male	650 (49.2)	557 (58.2)		
Female	671 (50.8)	400 (41.7)		
BMI subgroup			8.242	0.041
Slimmer	81 (6.1)	39 (4.0)		
Normal	734 (55.5)	522 (54.5)		
Overweight	378 (28.6)	313 (32.7)		
Obesity	128 (9.6)	83 (8.6)		
Age subgroup			203.499	<0.001
18–45	639 (48.3)	201 (21.0)		
45–59	416 (31.4)	363 (37.9)		
>59	266 (20.1)	393 (41.0)		
Duration of residence in Wuxi County (year)			101.153	<0.001
<5	47 (3.5)	8 (0.8)		
5–10	51 (3.8)	17 (1.7)		
10–20	162 (12.2)	55 (5.7)		
20–29	247 (18.6)	104 (10.8)		
>29	814 (61.6)	773 (80.7)		
Educational level			214.798	<0.001
Primary and below	417 (31.5)	550 (57.4)		
Senior high school	227 (17.1)	198 (20.6)		
Above high school	677 (51.2)	209 (21.8)		
Occupation			267.739	<0.001
Farmer	427 (32.3)	607 (63.4)		
Worker	121 (9.1)	72 (7.5)		
Civil servant	401 (30.3)	80 (8.3)		
Self-employed	72 (5.4)	57 (5.9)		
Service Industry	91 (6.8)	53 (5.5)		
Other occupation	209 (15.8)	88 (9.1)		
Smoking^b^			13.703	<0.001
No smoking	907 (68.6)	573 (59.8)		
Previous smoking	87 (6.5)	57 (5.9)		
Always smoking.	319 (24.1)	288 (30.0)		
Alcohol consumption^c^			19.159	<0.001
No drinking	724 (54.8)	578 (60.3)		
Sometimes	355 (26.8)	132 (13.7)		
Previous drinking	75 (5.6)	39 (4.0)		
Always drinking	148 (11.2)	161 (16.8)		
Chronic disease				
Hypertension	247 (18.6)	156 (16.3)	7.233	0.007
Diabetes mellitus	163 (12.3)	27 (2.8)	0.001	0.977
Hyperlipidemia	37 (2.8)	14 (1.4)	13.204	<0.001
Lung disease	25 (1.8)	20 (2.0)	0.112	0.738
stroke	12 (0.9)	11 (1.1)	0.323	0.570
Heart disease	15 (1.1)	8 (0.8)	0.498	0.480
Liver disease	10 (0.7)	8 (0.8)	0.044	0.834
Cancer	5 (0.3)	3 (0.3)	0.067	0.796
Kidney disease	6 (0.4)	2 (0.2)	0.954	0.329

^a^Those who had consumed Hawk tea for >10 years, with a frequency of >3 times per week and >2 cups per serving were defined as heavy drinkers.

^b^Forty-seven people did not provide smoking information.

^c^Sixty-six people did not provide alcohol consumption information.

### 3.3 Reported discomforts after drinking hawk tea

As shown in Table 3, among the 2,278 people who were habitual drinkers of hawk tea, 2.7% (62 people) reported that they had experienced at least more than one type of slight discomfort after drinking hawk tea. Among the discomforts reported, symptoms which was not clearly defined with a specific category had the proportion of 67.7%, such as thirst, irritability, headache, dizziness, panic, sweating, followed by digestive symptom (35.5%), dermal and mucous membrane symptoms (6.5%) and respiratory symptoms (3.2%) (Table 3).

**Table 3:** Discomfort experiences reported after drinking hawk tea (%).

Adverse reactions	Frequency (*N* = 70^a^)	Proportion (%)
Digestive symptoms^b^	22	35.5%
Dermal and mucous membrane symptoms^c^	4	6.5%
Respiratory symptoms^d^	2	3.2%
Other symptoms^e^	42	67.7%

^a^Some participants reported more than one symptom.

^b^Digestive symptoms (nausea, vomiting, abdominal distension, diarrhea, abdominal pain, and poor appetite).

^c^Dermal and mucous membrane symptoms (itchy skin hyperpigmentation, skin seeds and pimples, pallor, bruising of the lips, and red itchy eyes).

^d^Respiratory symptoms (itchy, runny, stuffy nose, cough, shortness of breath, difficulty in breathing).

^e^Other symptoms (thirst, irritability, headache, dizziness, panic, and sweating).

### 3.4 Univariate analysis of the factors related to discomfort experiences

Except for those who lived in WuXi for > 29 years and had a lower risk of adverse reactions compared to others, the discomfort experience was not associated with other factors, such as sex, age, BMI, education level, occupation, smoking status, and alcohol consumption (Table 4).

**Table 4:** Univariate analysis of discomfort experience of drinking hawk tea.

Characteristics	Have any discomfort experience (*N* = 62)	No discomfort experienced (*N* = 2,216)	OR^a^ (95% CI^b^)	*p*-value
Sex				
Male	39	1,168	1.00	
Female	23	1,048	0.66 (0.39–1.11)	0.115
BMI				
Slimmer	4	116	1.00	
Normal	35	1,221	0.83 (0.29–2.38)	0.731
Overweight	14	677	0.60 (0.19–1.85)	0.375
Obesity	9	202	1.29 (0.39–4.29)	0.675
Age				
18–45	25	815	1.00	
45–59	19	760	0.82 (0.45–1.49)	0.507
>59	18	641	0.92 (0.50–1.69)	0.778
Duration of residence (year)				
<5	4	51	1.00	
5–10	3	65	0.59 (0.13–2.75)	0.500
10–20	5	212	0.30 (0.08–1.16)	0.081
20–29	10	341	0.37 (0.11–1.24)	0.107
>29	40	1,547	0.33 (0.11–0.96)	0.041
Educational level				
Primary and below	20	947	1.00	
Senior high school	14	411	1.61 (0.81–3.22)	0.176
Above high school	28	858	1.55 (0.86–2.76)	0.142
Occupation			1.03 (0.89–1.18)	0.723
Farmer	25	1,009	1.00	
Worker	6	187	1.29 (0.52–3.20)	0.575
Civil servant	15	466	1.30 (0.68–2.49)	0.430
Self-employed	3	126	0.96 (0.29–3.23)	0.949
Service Industry	7	137	2.06 (0.88–4.86)	0.098
Other occupation	6	291	0.83 (0.34–2.05)	0.689
Smoking situation^c^				
No smoking	36	1,444	1.00	
Previous smoking	6	138	1.74 (0.72–4.21)	0.216
Always smoking.	12	595	0.81 (0.42–1.57)	0.529
Alcohol consumption^d^				
No drinking	30	1,272	1.00	
Sometimes	16	471	1.44 (0.78–2.67)	0.246
Previous drinking	3	111	1.15 (0.34–3.81)	0.824
Always drinking	2	307	0.28 (0.07–1.16)	0.079
Chronic disease				
Hypertension	8	311	0.91 (0.43–1.93)	0.800
Diabetes mellitus	1	63	0.56 (0.08–4.10)	0.568
Hyperlipidaemia	3	65	1.68 (0.51–5.51)	0.390
Lung disease	0	45	NA	NA
Stroke	1	22	1.63 (0.22–12.33)	0.633
Heart disease	1	22	1.63 (0.22–12.33)	0.633
Liver disease	0	18	NA	NA
Cancer	0	8	NA	NA
Kidney disease	0	8	NA	NA

^a^OR = odds ratio.

^b^95%CI = 95% confidence interval.

^c^Eight people did not provide smoking information.

^d^Eleven people did not provide alcohol consumption information.

### 3.5 The hawk tea consumption behavior and discomfort experience

In the multivariate logistic regression analysis, hawk tea drinking duration, frequency, volume, and drinking status were not significantly associated with discomfort experiences, even after adjusting for all available covariates (all *p*-values > 0.05) (Table 5).

**Table 5:** Logistic analysis of hawk tea consumption and experience of discomforts.

Item	Unadjusted OR^b^ (95%CI^c^)	*p*-value	Adjusted^a^ OR^b^ (95%CI^c^)	*p*-value
Drinking duration (year)				
<5	1		1	
5–10	0.51 (0.22–1.19)	0.117	0.49 (0.20–1.21)	0.122
>10	0.66 (0.38–1.14)	0.135	0.60 (0.29–1.24)	0.169
Drinking frequency (time/weekly)				
1–3	1		1	
3–5	0.76 (0.35–1.65)	0.490	0.77 (0.34–1.75)	0.534
>5	1.23 (0.68–2.24)	0.498	1.47 (0.68–3.15)	0.325
Drinking volume (cup/per serving)				
1–2	1		1	
2–3	1.12 (0.61–2.06)	0.703	1.21 (0.62–2.40)	0.576
>3	0.88 (0.43–1.77)	0.717	0.48 (0.18–1.32)	0.154
Drinking status				
Light drinkers	1		1	
Heavy drinkers	1.22 (0.74–2.02)	0.442	1.16 (0.61–2.20)	0.652

^a^Adjusted for sex, age, BMI, occupation, education, duration of residence, smoking status, alcohol consumption, and chronic diseases.

^b^OR = odds ratio.

^c^95%CI = 95% confidence interval.

### 3.6 Hawk tea consumption and chronic diseases

**Figure 2:**
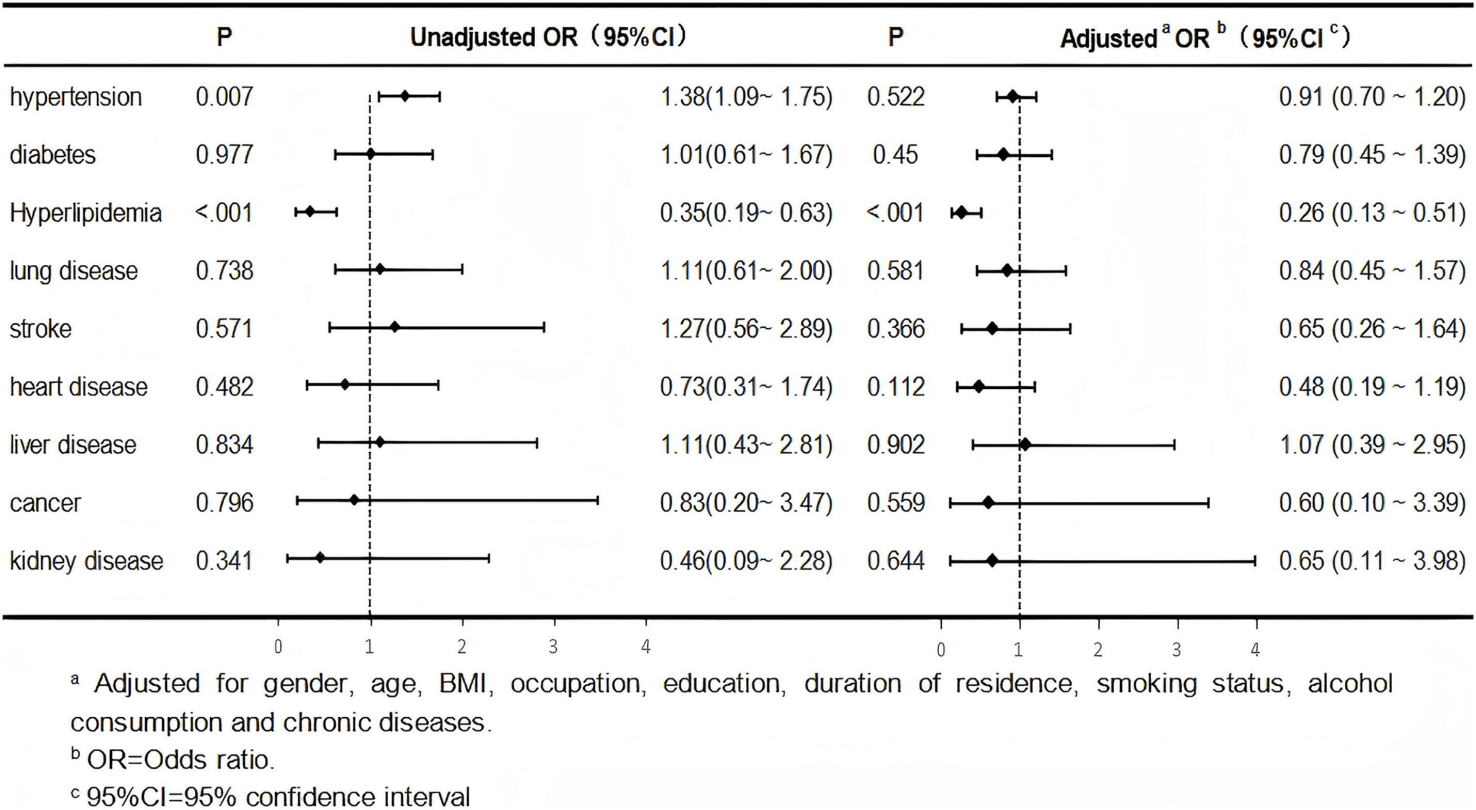
Multivariate logistic-analysis of the risks of chronic diseases in heavy drinkers compared to light drinkers.

After adjusting for confounders such as sex, age, BMI, occupation, education, local residence time, smoking status, and alcohol consumption, we found that Heavy Hawk tea drinkers had a lower risk of hyperlipidaemia than light drinkers (*p* < 0.001, odds ratio = 0.26), and no statistically significant differences were noted in the risk of other chronic diseases between heavy and light drinkers (all adjusted *p*-value >0. 05) (Figure 2).

## 4 Discussion

In this study, we found that consumption of hawk tea was highly prevalent in Wuxi County, with more than half of the samples drinking hawk tea more than once a month, and 55.2% of the drinkers had a history of drinking it for > 10 years. This indicated that the habitual drinking of hawk tea was more common among local residents.

Regarding the safety of drinking Hawk tea, multivariate logistic regression revealed no significant association between the occurrence of discomfort symptoms and the duration, frequency, or amount of Hawk tea consumption. The lack of a dose–response (in terms of duration, frequency, and amount) of adverse effects suggests that these uncomfortable reactions may be induced by chance with other unknown stimulators or health conditions, rather than by the consumption of hawk tea. The toxicological studies on high dose of total flavonoids extract of hawk tea leaves on rats showed no observed side effects (8). Saponin which are rich in hawk tea have been reported to induce some acute symptoms in cold blooded animal, but not in mammals (20). Because we cannot identify the relationships between adverse reactions and any the drinking characteristics in this study, the possible compounds which may responsible for these reported discomforts was not clear, and allergic reaction to the ingredients of hawk tea may be possible and cannot be excluded.

We also found that hawk tea consumption did not increase the risk of developing common chronic diseases. In contrast, hawk tea consumption for >10 years, more than three times a week, and more than two cups each time, which we defined as heavy drinking, reduced the risk of hyperlipidaemia. The protective effect of hawk tea on hyperlipidaemia is consistent with the findings of Feng et al. (21), who reported that *L. coreana* extract (HTE) induced the transcription of the low-density lipoprotein receptor by inhibiting free cholesterol uptake. It also reduces the production of very low-density lipoproteins.

Regarding the mechanisms of the benefits of hawk tea, the flavonoids and polyphenols in hawk tea have the features of antioxidant (22–25), anti-inflammatory (26, 27) and other biological activities (28). These substances scavenge free radicals and inhibit the inflammatory response. Volatile oil components in hawk tea inhibit *Escherichia coli* and *Staphylococcus aureus* (29, 30). Hawk tea also contains saponins (31, 32), which may promote digestion and relieve gastrointestinal dyskinesia. More specific, total flavonoids extracts of hawk tea was found increased the expression of peroxisome proliferator-activated receptor alpha (PPARalpha) in high fat diet fed rat liver. These benefits were associated with increased superoxide dismutase (SOD) and decreased malondialdehyde (MDA) in high fat diet fed rat liver (33). Metabolic profile analysis identified dozens of metabolites which can adjust pathways of MAPK and PI3K/AKT to exert antioxidant activity of hawk tea (34). Secondly, extracts from hawk tea can suppress the IRE1/mTORC1/TNF-*α*-regulated inflammatory response initiated in peritoneal macrophages, exerting anti-inflammatory and immunomodulatory effects (35). In addition, extracts of hawk tea can down regulate the expression of adipose differentiation-related protein (ADRP) in the liver and improve the lipids profiles (36).

The strength of this study is that the sample size was large and the consumption of hawk tea was popular at the study site, which allowed us to detect the related adverse events and health effects. A limitation of this study is its cross-sectional design with self-reported information; the measurement of tea consumption, health outcomes, and confounders may have been biased by recall, and some other confounders were not included.

## 5 Conclusion

Long-term regular consumption of hawk tea has little chance of resulting in any recognizable acute adverse effects on consumers. The self-reported discomforts after drinking hawk tea seem to be coincidence with other symptom inducers. If discomfort symptom occur after drinking, temporary isolation from hawk tea may help. Besides, habitually drinking hawk tea may benefit lipid metabolism. Hawk tea can be generally recognized as a safe herbal beverage with health functions. However, further intervention studies are warranted to further confirm its function and safety in animal and population studies.

## Data Availability

The raw data supporting the conclusions of this article will be made available by the authors, without undue reservation.
